# Evaluation of spectroscopic properties of Er^3+^/Yb^3+^/Pr^3+^: SrGdGa_3_O_7_ crystal for use in mid-infrared lasers

**DOI:** 10.1038/srep13988

**Published:** 2015-09-15

**Authors:** Houping Xia, Jianghe Feng, Yan Wang, Jianfu Li, Zhitai Jia, Chaoyang Tu

**Affiliations:** 1 Key Laboratory of Optoelectronic Materials Chemistry and Physics, Fujian Institute of Research on the Structure of Matter, Chinese Academy of Science, Fuzhou 350002, PR China; 2 University of Chinese Academy of Sciences, Beijing 100039, PR China; 3 State Key Laboratory of Structural Chemistry, Fujian Institute of Research on the Structure of Matter, Chinese Academy of Sciences, Fuzhou 350002, PR China; 4 State Key Laboratory of Crystal Materials, Shandong University, Ji’nan 250100, PR China

## Abstract

Er^3+^/Yb^3+^/Pr^3+^: SrGdGa_3_O_7_ crystal was firstly grown by Czochralski method. Detailed spectroscopic analyses of Er^3+^/Yb^3+^/Pr^3+^: SrGdGa_3_O_7_ were carried out. Besides better absorption characteristic, the spectra of Er^3+^/Yb^3+^/Pr^3+^: SrGdGa_3_O_7_ show weaker up-conversion and near-infrared emissions as well as superior mid-infrared emission in comparison to Er^3+^: SrGdGa_3_O_7_ and Er^3+^/Yb^3+^: SrGdGa_3_O_7_ crystals. Furthermore, the self-termination effect for Er^3+^ 2.7 *μ*m laser is suppressed successfully because the fluorescence lifetime of the ^4^I_13/2_ lower level of Er^3+^ decreases markedly while that of the upper ^4^I_11/2_ level changes slightly in Er^3+^/Yb^3+^/Pr^3+^: SrGdGa_3_O_7_ crystal. The sensitization effect of Yb^3+^ and deactivation effect of Pr^3+^ ions as well as the energy transfer mechanism in Er^3+^/Yb^3+^/Pr^3+^: SrGdGa_3_O_7_ crystal were also studied in this work. The introduction of Yb^3+^ and Pr^3+^ is favorable for achieving an enhanced 2.7 *μ*m emission in Er^3+^/Yb^3+^/Pr^3+^: SrGdGa_3_O_7_ crystal which can act as a promising candidate for mid-infrared lasers.

In recent years, novel mid-infrared lasers with outputs in the range of 2.7–3 *μ*m have received extensive attention owing to enormous potential applications in medicine, sensing, light detection, *etc*.^1^,^2^,^3^ Er^3+^ can act as active ions achieving 2.7–3 *μ*m lasers through its ^4^I_11/2_ → ^4^I_13/2_ transition, which has been extensively investigated. However, a detrimental self-termination bottleneck effect is possible for this transition, because of the shorter lifetime of upper laser level (^4^I_11/2_) than that of the lower one (^4^I_13/2_). Previous researches have shown that increasing Er^3+^ concentration or co-doping with proper sensitive ions (such as Pr^3+^, Ho^3+^, Tm^3+^, *etc*.) can inhibit the self-termination bottleneck^4^,^5^,^6^,^7^,^8^,^9^,^10^. However, high doping concentration of Er^3+^ may lead to poor quality of crystal. So co-doping with proper sensitive ions is a better choice. Additionally, Yb^3+^ can also be introduced into Er^3+^-doped materials to improve absorption properties because of effective energy transfer between Yb^3+^ and Er^3+^ 11,12,13,14. Predictably, Er^3+^, Re^3+^ (Re^3+^ = Pr^3+^, Ho^3+^, Tm^3+^, *etc*.) and Yb^3+^ triply doped crystals without heavily doping of Er^3+^ might demonstrate excellent mid-infrared lasers performance. For example, Chen *et al*. developed a novel mid-infrared crystal Er^3+^/Yb^3+^/Ho^3+^: GYSGG^15^ in which Yb^3+^ can broaden the absorption band as well as shift the pumping peak to the optimum position, meanwhile, Ho^3+^ ions can nearly retain the lifetime of ^4^I_11/2_ level and decrease that of ^4^I_13∕2_ level intensely. Therefore, Er^3+^, Yb^3+^ and Re^3+^ (Re^3+^ = Pr^3+^, Ho^3+^, Tm^3+^, *etc*.) triply doped crystals are worth studying as mid-infrared lasers candidates.

Among the various matrix materials of mid-infrared solid-state lasers, fluoride, aluminate and gallate crystals are excellent for their low phonon energy, high thermal stability, stable chemical durability and so on^4^,^5^,^16^,^17^. The spectroscopic properties and laser operations of Er^3+^ doped CaF_2_, LiYF_4_, Y_3_Al_5_O_12_, CaYAlO_4_, CaGdAlO_4_, YSGG, GSGG and GGG crystals have been reported^4^,^5^,^17^,^18^,^19^,^20^,^21^. In this work, SrGdGa_3_O_7_ single crystal was chosen as the host matrix because it has lower melting point (about 1600 °C)^22^ as compared with the above aluminate and gallate crystals, and, large-sized crystals with high optical quality can be obtained more easily by using Czochralski technique.

SrGdGa_3_O_7_ single crystal belongs to the large family ABGa_3_O_7_ (A = Ca, Sr, Ba; B = Gd, La)^22^. It crystallizes in *P*
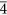
2_1_*m* space group and features a layered structure (Fig. 1(a,b)). The Ga^3+^ ions exhibit two kinds of slight distorted tetrahedral environments hereafter referred to as Ga_1_ and Ga_2_. Ga_1_O_4_ and Ga_2_O_4_ tetrahedra are alternately interconnected via corner-sharing to form anionic layer of [Ga_3_O_7_]^5−^ in *ab*-plane. Between the layers, the Sr^2+^ and Gd^3+^ cations are distributed randomly in the ratio of 1:1, which results in certain structural disorder. Therefore, the absorption and emission lines of Er^3+^ ions would be inhomogeneously broadened in Er^3+^ ions doped SrGdGa_3_O_7_ crystal. The broadening of absorption band is favorable for diode pumping and the broadening of emission band is very beneficial for tunable or ultra fast laser output. In addition, the Raman spectrum in Fig. 1(c) shows that SrGdGa_3_O_7_ single crystal has relative low phonon energy (~680 nm^−1^), which is similar to its isostructural SrLaGa_3_O_7_ crystal^23^. The low phonon energy conduces to the decrease of the multiphonon relaxation rate for Er^3+^: ^4^I_11/2_ → ^4^I_13/2_ transition, and therefore decreasing the populations on ^4^I_13/2_ state^12^,^24^,^25^.

This work aims to study the spectroscopic performance with focus on 2.7 *μ*m emissions and the roles of Yb^3+^ and Pr^3+^ in Er^3+^/Yb^3+^/Pr^3+^: SrGdGa_3_O_7_ crystal. To the best of our knowledge, Yb^3+^, Er^3+^ and Pr^3+^ triply doped single crystal was firstly investigated.

## Materials and Methods

Er^3+^/Yb^3+^/Pr^3+^: SrGdGa_3_O_7_ single crystal was successfully grown by Czochralski method. The initial concentrations of Er^3+^, Yb^3+^ and Pr^3+^ were 15 at.%, 5 at.% and 0.15 at.%, respectively. 15 at.% Er^3+^/5 at.% Yb^3+^: SrGdGa_3_O_7_, 15 at.% Er^3+^: SrGdGa_3_O_7_ and 5 at.% Yb^3+^: SrGdGa_3_O_7_ single crystals were also grown for comparison. The used chemicals were SrCO_3_ (A.R. grade) and Gd_2_O_3_, Er_2_O_3_, Yb_2_O_3_, Pr_2_O_3_, Ga_2_O_3_ (4N purity). In order to compensate the evaporation losses of Ga_2_O_3_ in the growing process, excess 1.0 wt.% Ga_2_O_3_ was added to the starting materials. The crystal growth was carried out in a DJL-400 furnace with N_2_ atmosphere protection, and an Ir crucible with the diameter of 50 mm and the height of 30 mm was used. A <001> orientated SrGdGa_3_O_7_ seed crystal was used. The pulling rate varied from 1 to 1.5 mm/h and the crystal rotation speed was kept at 8–15 r/m. Finally, the crystal was cooled to room temperature at a rate of 10–25 K/h.

The concentrations of Er^3+^, Yb^3+^ or Pr^3+^ ions in every crystal were measured by using an inductively coupled plasma atomic emission spectrometry (ICP-AES, Ultima2, Jobin-Yvon). The absorption spectra were measured using a Perkin-Elmer UV–VIS–NIR Spectrometer (Lambda-900). The emission spectra and fluorescence lifetime were measured using an Edinburgh Instruments Fluorescence Spectrometer. Samples with dimensions of 10.0 × 10.0 × 0.9 mm^3^ were optically polished for spectral measurement. The experimental conditions were maintained exactly same for measurement of each group of spectra in order to get the comparable results.

## Results and Discussion

### Ions concentrations

The concentrations of Er^3+^, Yb^3+^ and Pr^3+^ ions in Er^3+^/Yb^3+^/Pr^3+^: SrGdGa_3_O_7_ crystal are measured to be 5.06 × 10^20^ ions·cm^−3^, 1.08 × 10^20^ ions·cm^−3^ and 0.36 × 10^20^ ions·cm^−3^, respectively. The concentrations of Er^3+^ and Yb^3+^ ions in Er^3+^/Yb^3+^: SrGdGa_3_O_7_ crystal are 4.83 × 10^20^ ions·cm^−3^ and 1.04 × 10^20^ ions·cm^−3^, respectively. While the Er^3+^ concentration in Er^3+^: SrGdGa_3_O_7_ crystal is 4.86 × 10^20^ ions·cm^−3^ and Yb^3+^ concentration in Yb^3+^: SrGdGa_3_O_7_ crystal is 1.12 × 10^20^ ions·cm^−3^.

### Absorption spectra

**Figure 2:**
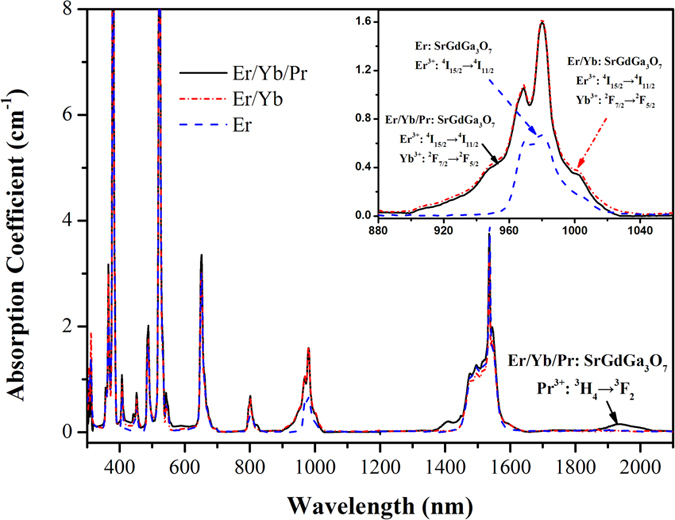
Absorption spectra of Er^3+^: SrGdGa_3_O_7_, Er^3+^/Yb^3+^: SrGdGa_3_O_7_ and Er^3+^/Yb^3+^/Pr^3+^: SrGdGa_3_O_7_ crystals at room temperature.

Room temperature absorption spectra of Er^3+^: SrGdGa_3_O_7_, Er^3+^/Yb^3+^: SrGdGa_3_O_7_ and Er^3+^/Yb^3+^/Pr^3+^: SrGdGa_3_O_7_ crystals are shown in Fig. 2. For Er^3+^: SrGdGa_3_O_7_ crystal, the absorption spectrum consists of six main absorption bands centered at 487, 522, 651, 802, 980 and 1536 nm, which correspond to the transitions from ^4^I_15/2_ to ^4^F_7/2_, ^2^H_11/2_, ^4^F_9/2_, ^4^I_9/2_, ^4^I_11/2_ and ^4^I_13/2_, respectively. Among them the wide absorption band centered at 980 nm with a full width at half maximum (FWHM) of 31 nm matches the commercial 980 nm InGaAs laser diodes (980 nm LDs) very well. The absorption cross-section at 980 nm is calculated to be 2.1 × 10^−21^ cm^2^. As compared with the absorption spectrum of Er^3+^: SrGdGa_3_O_7_ crystal, there is almost no change in the intensity and location of the characteristic absorption bands of Er^3+^ ions in Er^3+^/Yb^3+^: SrGdGa_3_O_7_ crystal. But, there is a much stronger absorption band in the range of 936 to 1015 nm with a FWHM of 40 nm, which is involved both the contributions from Yb^3+^ and Er^3+^ ions, owing to the transition from ^2^F_7∕2_ to ^2^F_5∕2_ of Yb^3+^ ions and the transition from ^4^I_15∕2_ to ^4^I_11∕2_ of Er^3+^ ions (Inset of Fig. 2). Its absorption peak is also located at 980 nm and the absorption cross-section is calculated to be 3.6 × 10^−21^ cm^2^. Thus, the introduction of Yb^3+^ ions not only enhanced absorption intensity but also widened the absorption band, which makes Er^3+^/Yb^3+^: SrGdGa_3_O_7_ crystal more suitable for 980 nm LDs pumping. Furthermore, as seen in the absorption spectrum of Er^3+^/Yb^3+^/Pr^3+^: SrGdGa_3_O_7_ crystal in the inset of Fig. 2, the absorption band centered at 980 nm is very similar to that in Er^3+^/Yb^3+^: SrGdGa_3_O_7_ crystal owing to the existence of Yb^3+^ ions in both crystals. For Pr^3+^ ions, a weak characteristic absorption band corresponding to the transition from ^3^H_4_ to ^3^F_2_ of Pr^3+^ ions is observed.

### Emission spectra

**Figure 3:**
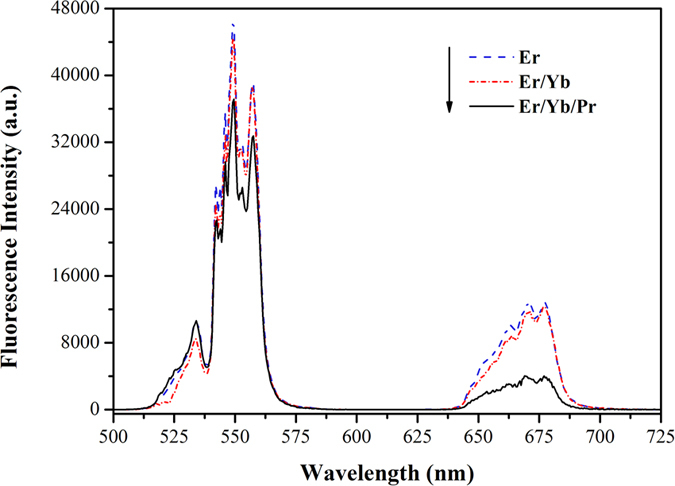
Up-conversion emission spectra of Er^3+^: SrGdGa_3_O_7_, Er^3+^/Yb^3+^: SrGdGa_3_O_7_ and Er^3+^/Yb^3+^/Pr^3+^: SrGdGa_3_O_7_ crystals under excitation of 980 nm.

The up-conversion emission spectra within 500–725 nm of Er^3+^: SrGdGa_3_O_7_, Er^3+^/Yb^3+^: SrGdGa_3_O_7_ and Er^3+^/Yb^3+^/Pr^3+^: SrGdGa_3_O_7_ crystals are measured under excitation of 980 nm, as shown in Fig. 3. In the spectra of Er^3+^: SrGdGa_3_O_7_ crystal, the observable green and red up-conversion emission bands which centered at 550 and 677 nm are assigned to Er^3+^: (^2^H_11/2_, ^4^S_3/2_) → ^4^I_15/2_ and ^4^F_9/2_ → ^4^I_15/2_ transitions, respectively. Compared with Er^3+^: SrGdGa_3_O_7_ crystal, the up-conversion emission spectrum of Er^3+^/Yb^3+^: SrGdGa_3_O_7_ crystal shows tiny changes in intensity while that of Er^3+^/Yb^3+^/Pr^3+^: SrGdGa_3_O_7_ crystal exhibits much weaker intensity.

**Figure 4:**
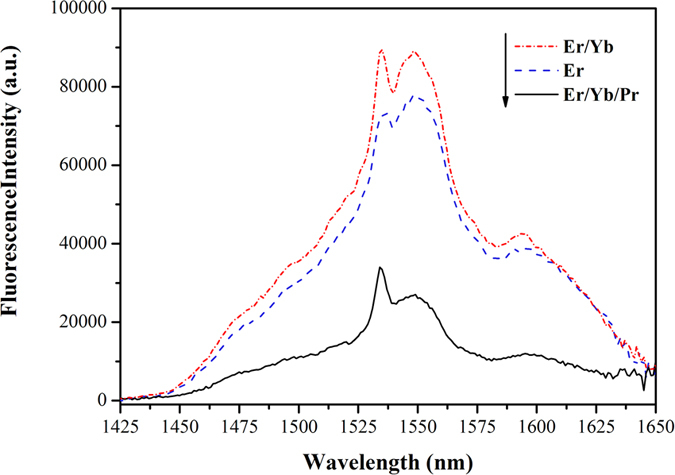
Near-infrared emission spectra of Er^3+^: SrGdGa_3_O_7_, Er^3+^/Yb^3+^: SrGdGa_3_O_7_ and Er^3+^/Yb^3+^/Pr^3+^: SrGdGa_3_O_7_ crystals.

The near-infrared emission spectra of three crystals within the range of 1425–1650 nm excited by 980 nm are shown in Fig. 4. For Er^3+^/Yb^3+^: SrGdGa_3_O_7_ crystal, the intensity of the 1553 nm emission corresponding to Er^3+^: ^4^I_13/2_ → ^4^I_15/2_ is stronger than that of Er^3+^ single-doped SrGdGa_3_O_7_. Generally, the strong near-infrared emission is a hindering factor for 2.7 *μ*m laser output, thus the sole introduction of Yb^3+^ into Er^3+^: SrGdGa_3_O_7_ crystal is not beneficial to the realization of 2.7 *μ*m lasers. However, the addition of Pr^3+^ can significantly inhibit the harmful near-infrared emission as shown in the spectra of Er^3+^/Yb^3+^/Pr^3+^: SrGdGa_3_O_7_ crystal.

**Figure 5:**
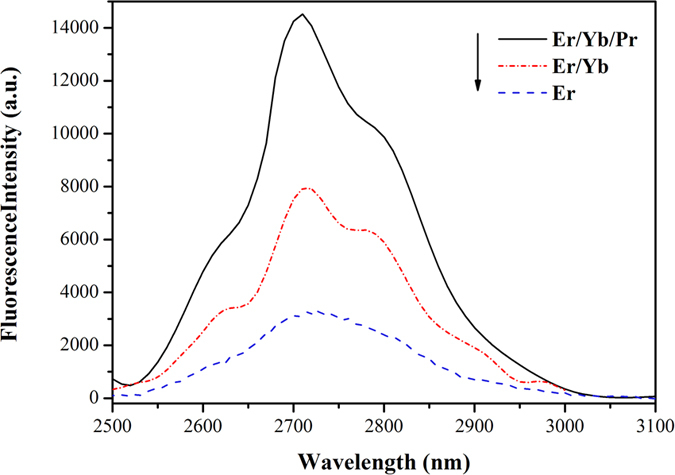
Mid-infrared emission spectra of Er^3+^: SrGdGa_3_O_7_, Er^3+^/Yb^3+^: SrGdGa_3_O_7_ and Er^3+^/Yb^3+^/Pr^3+^: SrGdGa_3_O_7_ crystals.

Figure 5 displays the mid-infrared emission spectra in the range of 2500–3100 nm excited by an optical parametric oscillator (OPO) laser at 980 nm. The broad mid-infrared emission band centered at 2718 nm can be assigned to the ^4^I_11/2_ → ^4^I_13/2_ transition of Er^3+^. There is almost no difference in the emission spectrum shape and peak position of the three crystals. However, it is obvious that the strongest 2.7 *μ*m emission existed in Er^3+^/Yb^3+^/Pr^3+^: SrGdGa_3_O_7_ crystal. The intensity is about 5 times of that in Er^3+^: SrGdGa_3_O_7_ crystal. The enhanced mid-infrared emission is also obtained in Er^3+^/Yb^3+^: SrGdGa_3_O_7_ crystal. As an important parameter affecting the potential 2.7 *μ*m laser performance, the stimulated emission cross-section, can be calculated by the following equation^5^,^26^:


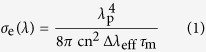


where *λ*_p_ is the emission peak wavelength, *c* is the velocity of light, *n* is the refractive index which can be obtained in^27^, *λ*_eff_ is the effective line width of the emission band^11^,^26^ and *τ*_m_ is the measured lifetime. The obtained emission cross-sections of Er^3+^: SrGdGa_3_O_7_, Er^3+^/Yb^3+^: SrGdGa_3_O_7_ and Er^3+^/Yb^3+^/Pr^3+^: SrGdGa_3_O_7_ crystals are all listed in Table 1.

### Fluorescence lifetimes

**Figure 6:**
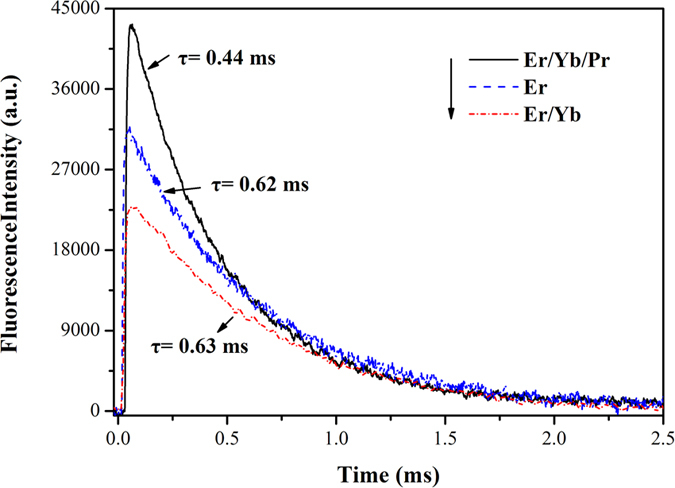
Decay curves of Er^3+^: ^4^I_11/2_ level in Er^3+^: SrGdGa_3_O_7_, Er^3+^/Yb^3+^: SrGdGa_3_O_7_ and Er^3+^/Yb^3+^/Pr^3+^: SrGdGa_3_O_7_ crystals.

**Figure 7:**
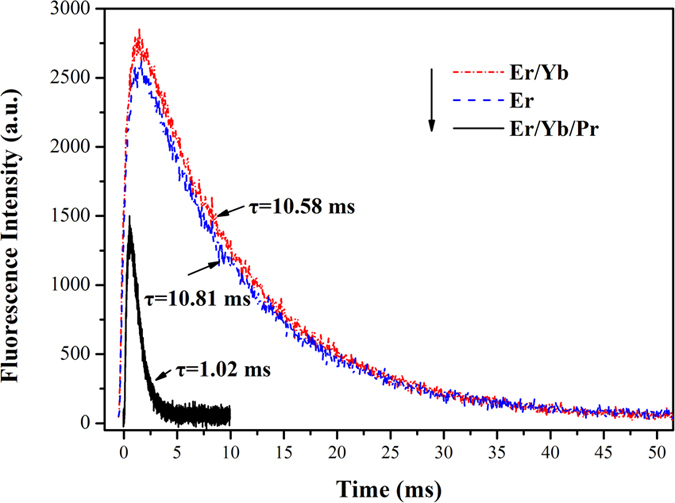
Decay curves of Er^3+^: ^4^I_13/2_ level in Er^3+^: SrGdGa_3_O_7_, Er^3+^/Yb^3+^: SrGdGa_3_O_7_ and Er^3+^/Yb^3+^/Pr^3+^: SrGdGa_3_O_7_ crystals.

The decay curves of Er^3+^: ^4^I_11/2_ and ^4^I_13/2_ levels in Er^3+^: SrGdGa_3_O_7_, Er^3+^/Yb^3+^: SrGdGa_3_O_7_ and Er^3+^/Yb^3+^/Pr^3+^: SrGdGa_3_O_7_ crystals are shown in Fig. 6 and 7, respectively. For Er^3+^: SrGdGa_3_O_7_ crystal, the decay curves both show single exponential decaying behavior and the fluorescence lifetimes can be fitted with


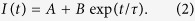


However, the decay curves of ^4^I_11/2_ and ^4^I_13/2_ levels of Er^3+^ in Er^3+^/Yb^3+^: SrGdGa_3_O_7_ and Er^3+^/Yb^3+^/Pr^3+^: SrGdGa_3_O_7_ crystals show multi-exponential behavior, which can be obtained by fitting with


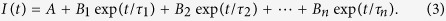


Then, the fluorescence lifetimes are calculated by


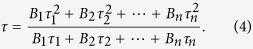


The fluorescence lifetimes are listed in Table 1, too. When compared with Er^3+^: SrGdGa_3_O_7_, the fluorescence lifetimes of ^4^I_11/2_ and ^4^I_13/2_ levels of Er^3+^ only change slightly in Er^3+^/Yb^3+^: SrGdGa_3_O_7_ crystal. However, the fluorescence lifetimes of ^4^I_11/2_ and ^4^I_13/2_ levels of Er^3+^ both decrease in Er^3+^/Yb^3+^/Pr^3+^: SrGdGa_3_O_7_ crystal, which is mainly due to energy transfer via Er^3+^: ^4^I_11/2_ → Pr^3+^: ^1^G_4_ and Er^3+^: ^4^I_13/2_ → Pr^3+^: ^3^F_4_. The declining degree of the lifetime of ^4^I_13/2_ level is much larger than that of ^4^I_11/2_ level. To be more specific, the lifetime of ^4^I_11/2_ level is reduced to 1/1.4 while the lifetime of ^4^I_13/2_ level is reduced to 1/10.6 as compared with that in Er^3+^: SrGdGa_3_O_7_ crystal. Based on the fluorescence lifetimes, the energy transfer efficiency from Er^3+^ to Pr^3+^ in Er^3+^/Yb^3+^/Pr^3+^: SrGdGa_3_O_7_ crystal can be obtained from the following equation^4^,^5^,^8^:


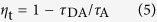


where *τ*_DA_ is the lifetime of Er^3+^ in the existence of Pr^3+^, and *τ*_A_ is the lifetime of Er^3+^ in the absence of Pr^3+^ 4. The calculated energy transfer efficiencies of Er^3+^: ^4^I_11/2_ → Pr^3+^: ^1^G_4_ and Er^3+^: ^4^I_13/2_ → Pr^3+^: ^3^F_4_ are about 29.0% and 90.6%, respectively. That means the lifetime of the Er^3+^: ^4^I_13/2_ level decreases much quicker than that of Er^3+^: ^4^I_11/2_ level in Er^3+^/Yb^3+^/Pr^3+^: SrGdGa_3_O_7_ crystal. Consequently, co-doping Pr^3+^ can significantly decrease the lifetime of the lower level (Er^3+^: ^4^I_13/2_) and almost has no effect on that of the upper level (Er^3+^: ^4^I_11/2_), which indicates the self-termination problem in the crystal can be greatly inhibited.

**Figure 8:**
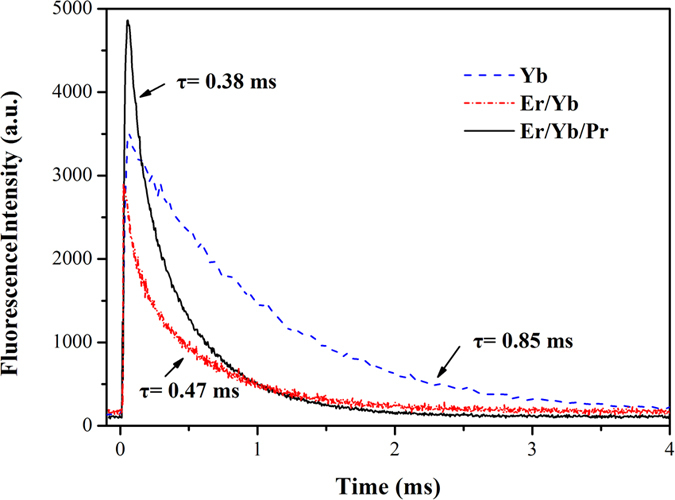
Decay curves of Yb^3+^: ^2^F_5/2_ level in Yb^3+^: SrGdGa_3_O_7_, Er^3+^/Yb^3+^: SrGdGa_3_O_7_ and Er^3+^/Yb^3+^/Pr^3+^: SrGdGa_3_O_7_ crystals.

To explore the sensitization effect of Yb^3+^ in SrGdGa_3_O_7_ crystals, the decay curves of Yb^3+^: ^2^F_5/2_ energy levels in Er^3+^/Yb^3+^: SrGdGa_3_O_7_, Er^3+^/Yb^3+^/Pr^3+^: SrGdGa_3_O_7_ and Yb^3+^: SrGdGa_3_O_7_ crystals are measured and shown in Fig. 8. For Yb^3+^: SrGdGa_3_O_7_ crystal, the decay curve is single exponential, while the decay curve of Yb^3+^: ^2^F_5/2_ in Er^3+^/Yb^3+^: SrGdGa_3_O_7_ crystal shows multi-exponential behavior. The fitted fluorescence lifetimes are also listed in Table 1. The energy transfer efficiency from Yb^3+^ ions to Er^3+^ ions can also be obtained from 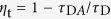, where *τ*_DA_ is the lifetime of Yb^3+^ ions in Er^3+^/Yb^3+^: SrGdGa_3_O_7_ crystal, *τ*_D_ is the lifetime of Yb^3+^ ions in Yb^3+^: SrGdGa_3_O_7_ crystal. The energy transfer efficiency is calculated to be 44.7%. For Er^3+^/Yb^3**+**^/Pr^3+^: SrGdGa_3_O_7_ crystal, the decay curve of Yb^3+^: ^2^F_5/2_ also shows multi-exponential behavior and the fitted fluorescence lifetime is shorter than that in Er^3+^/Yb^3+^: SrGdGa_3_O_7_ crystal. The corresponding energy transfer efficiency is calculated to be 55.3%. This suggests that the energy transfer between Yb^3+^ and Pr^3+^ exists in Er^3+^/Yb^3+^/Pr^3+^: SrGdGa_3_O_7_ crystal. Nevertheless, this small proportion of energy shows no obvious negative effect on the 2.7 *μ*m emission in Er^3+^/Yb^3+^/Pr^3+^: SrGdGa_3_O_7_ crystal, which is shown in above experimental results. In conclusion, Yb^3+^ is a practicable sensitizer to enhance the 2.7 *μ*m emission in Er^3+^/Yb^3+^/Pr^3+^: SrGdGa_3_O_7_ crystal, that is, Yb^3+^ ions can effectively transfer pumping energy to Er^3+^ ions and thus enhance the absorption efficiency of Er^3+^ ions obviously. Furthermore, higher Yb^3+^ concentrations may result in an increase in energy transfer efficiency between Yb^3+^ and Er^3+^ because the efficiency of energy transfer between Yb^3+^ and other ions partly depends on Yb^3+^ ions’ density^11^.

### Energy transfer diagram

**Figure 9:**
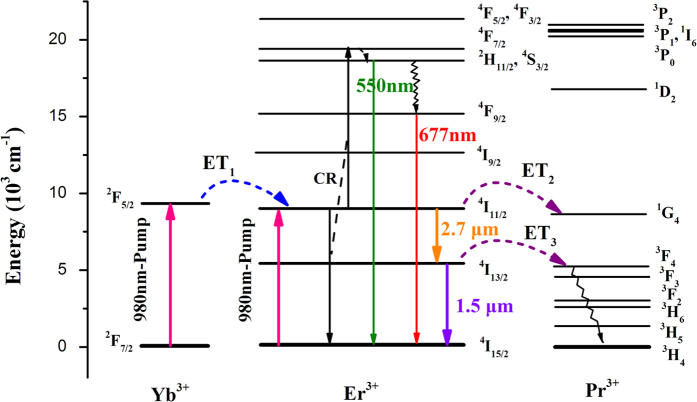
The energy transfer diagram among Yb^3+^, Er^3+^ and Pr^3+^ ions in Er^3+^/Yb^3+^/Pr^3+^: SrGdGa_3_O_7_ crystal.

Based on the analyses of measurements above, the possible energy transfer processes in Er^3+^/Yb^3+^/Pr^3+^: SrGdGa_3_O_7_ crystal can be interpreted by the energy transfer diagram in Fig. 9. When Er^3+^/Yb^3+^/Pr^3+^: SrGdGa_3_O_7_ crystal is pumped, populations on Yb^3+^: ^2^F_7/2_ level and populations on Er^3+^: ^4^I_15/2_ level are excited to Yb^3+^: ^2^F_5/2_ and Er^3+^: ^4^I_11/2_ levels by ground state absorption. Then an anticipated energy transfer progress Yb^3+^: ^2^F_5/2_ → Er^3+^: ^4^I_11/2_ (ET_1_) takes place and increases the populations on Er^3+^: ^4^I_11/2_ level, which leads to an enhanced 2.7 *μ*m emission. Although the enhanced 2.7 *μ*m emission can increase the populations on Er^3+^: ^4^I_13/2_ level subsequently, the competitive near-infrared emission is still weakened because of the effective energy transfer Er^3+^: ^4^I_13/2_ → Pr^3+^: ^3^F_4_ (ET_3_) which deactivates the population on the Er^3+^: ^4^I_13/2_ state effectively. Furthermore, with the decrease of populations on the Er^3+^: ^4^I_11/2_ state as a result of the enhanced 2.7 *μ*m emission and the energy transfer progress Er^3+^: ^4^I_11/2_ → Pr^3+^: ^1^G_4_ (ET_2_), the cross-relaxation ^4^I_11/2_ + ^4^I_11/2_ → ^4^F_7/2_ + ^4^I_15/2_ (CR) from the Er^3+^: ^4^I_11/2_ state decreases. Consequently, the red and green up-conversion emissions are decreased significantly. Further, the decrease of near-infrared and up-conversion emission is beneficial to the 2.7 *μ*m emission conversely. So the enhanced 2.7 *μ*m emission is a result of comprehensive effect of above progresses.

## Conclusion

Er^3+^/Yb^3+^/Pr^3+^: SrGdGa_3_O_7_ crystal was grown successfully by Czochralski method, Er^3+^/Yb^3+^: SrGdGa_3_O_7_ and Er^3+^ and Yb^3+^ singly doped SrGdGa_3_O_7_ crystals were also grown for spectral comparison. The crystals were evaluated by absorption, up-conversion emission, near-infrared and mid-infrared emissions, and luminescence decay measurements. The relevant absorption and emission cross-sections as well as fluorescence lifetimes were calculated and compared. Studies showed that Yb^3+^ ions acted as a sensitizer for Er^3+^ due to the energy transfer via Yb^3+^: ^2^F_5/2_ → Er^3+^: ^4^I_11/2_ in Er^3+^/Yb^3+^/Pr^3+^: SrGdGa_3_O_7_ crystal, which increased the absorption cross-section with peak at 980 nm. At the same time, due to the existence of Pr^3+^, two energy transfer routes Er^3+^: ^4^I_11/2_ → Pr^3+^: ^1^G_4_ and Er^3+^: ^4^I_13/2_ → Pr^3+^: ^3^F_4_ occurred and the corresponding energy transfer efficiencies were determined to be 29.0% and 90.6%, respectively. As a result, strongest mid-infrared emissions as well as weakest up-conversion and near-infrared emissions were shown in Er^3+^/Yb^3+^/Pr^3+^: SrGdGa_3_O_7_ as compared with Er^3+^: SrGdGa_3_O_7_ and Er^3+^/Yb^3+^: SrGdGa_3_O_7_ crystals. Furthermore, the fluorescence lifetimes of Er^3+^: ^4^I_11/2_ and ^4^I_13/2_ levels both decreased in Er^3+^/Yb^3+^/Pr^3+^: SrGdGa_3_O_7_ crystal. But the decrease extent of the lifetime of lower level ^4^I_13/2_ is much larger than that of upper level ^4^I_11/2_. The lifetime of the ^4^I_13/2_ state decreases from 10.81 ms in Er^3+^: SrGdGa_3_O_7_ crystal to 1.02 ms in Er^3+^/Yb^3+^/Pr^3+^: SrGdGa_3_O_7_ crystal by 10 times. Obviously, the self-saturation for the Er^3+^ 2.7 *μ*m laser is inhibited to a great degree. These results indicate that the doping of Yb^3+^ and Pr^3+^ is helpful to achieve an enhanced 2.7 *μ*m emission and Er^3+^/Yb^3+^/Pr^3+^: SrGdGa_3_O_7_ crystal could be considered as an excellent candidate for mid-infrared lasers.

## Additional Information

**How to cite this article**: Xia, H. *et al*. Evaluation of spectroscopic properties of Er^3+^/Yb^3+^/Pr^3+^: SrGdGa_3_O_7_ crystal for use in mid-infrared lasers. *Sci. Rep*. **5**, 13988; doi: 10.1038/srep13988 (2015).

## Figures and Tables

**Figure 1:**
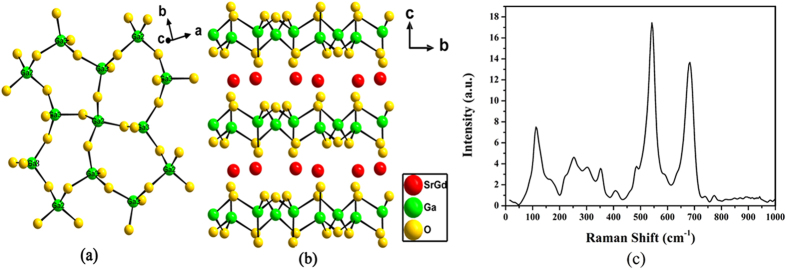
The view of anionic layer Ga2O (a), 3D network (b) and the Raman spectrum of SrGdGa_3_O_7_ (c).

**Table 1:** Spectroscopic data for Er^3+^: SrGdGa_3_O_7_, Er^3+^/Yb^3+^: SrGdGa_3_O_7_ and Er^3+^/Yb^3+^/Pr^3+^: SrGdGa_3_O_7_ laser crystals.

Crystals	*σ*_a_ at 980nm (10^−21^ cm^2^)	^4^I_11/2_, *σ*_e_ (10^−19^ cm^2^)	Er^3+^: ^4^I_13/2_		Er^3+^: ^4^I_11/2_		Yb^3+^: ^2^F_5/2_
			*τ*_f_ (ms)	*η*_t_ (ET_3_)	*τ*_f_ (ms)	*η*_t_ (ET_2_)	*τ*_f_(ms)
Er^3+^: SrGdGa_3_O_7_	2.1	1.64	10.81		0.62		
Er^3+^/Yb^3+^: SrGdGa_3_O_7_	3.5	1.75	10.58		0.63		0.47
Er^3+^/Yb^3+^/Pr^3+^: SrGdGa_3_O_7_	3.6	2.57	1.02	90.6%	0.44	29.0%	0.38
